# A Novel Algorithm for Detecting Microsatellite Instability Based on Next-Generation Sequencing Data

**DOI:** 10.3389/fonc.2022.916379

**Published:** 2022-06-30

**Authors:** Shijun Li, Bo Wang, Miaomiao Chang, Rui Hou, Geng Tian, Ling Tong

**Affiliations:** ^1^ Pathology Department, Chifeng Municipal Hospital, Chifeng, China; ^2^ Science Department, Geneis Beijing Co., Ltd., Beijing, China

**Keywords:** microsatellite instability (MSI), NGS, PCR-based methods, peak discovery, Smoothing

## Abstract

**Objectives:**

Microsatellite instability (MSI) is the condition of genetic hypermutability caused by spontaneous acquisition or loss of nucleotides during the DNA replication. MSI has been discovered to be a useful immunotherapy biomarker clinically. The main DNA-based method for MSI detection is polymerase chain reaction (PCR) amplification and fragment length analysis, which are costly and laborious. Thus, we developed a novel method to detect MSI based on next-generation sequencing (NGS) data.

**Methods:**

We chose six markers of MSI. After alignment and reads counting, a histogram was plotted showing the counts of different lengths for each marker. We then designed an algorithm to discover peaks in the generated histograms so that the peak numbers discovered in NGS data resembled that in PCR-based method.

**Results:**

We selected nine samples as the training dataset, 101 samples for validation, and 68 samples as the test dataset from Chifeng Municipal Hospital, Inner Mongolia, China. The NGS-based method achieved 100% accuracy for the validation dataset and 98.53% accuracy for the test dataset, in which only one false positive was detected.

**Conclusions:**

Accurate MSI judgments were achieved using NGS data, which could provide comparable MSI detection with the gold standard, PCR-based methods.

## Introduction

DNA mismatch repair (MMR) system is able to correct length-altering mutations during DNA replication. MMR dysfunction leads to insertion/deletion mutations in repeats of short non-coding microsatellites (1–6 bp). The spontaneous acquisition or loss of nucleotides in repetitive DNA sequence tracts is considered microsatellite instability (MSI). Inactive germline mutations in the MMR pathways (including MSH2, MSH6, MLH1, and PMS2) result in deficient MMR, which usually occurs in patients with Lynch syndrome (LS), and somatic promoter hypermethylation of MLH1 in the sporadic cancers (1, 2).

MSI analysis is useful in clinical implications for patients with colorectal cancer (CRC) such as classification of LS (2) and prediction of response to 5-fluorouracil–based adjuvant therapy (3), informing choice for immunotherapy and providing prognosis information (4–7). European Society for Medical Oncology has recommended MSI testing for better immunotherapy selection, and the National Comprehensive Cancer Network (NCCN) guidelines endorsed universal MSI or MMR testing for newly diagnosed CRC or endometrial cancer in 2018 to evaluate suspected patients with LS.

There are two most commonly used methods to determine MSI status, including immunohistochemistry (IHC) for MMR proteins and fluorescent multiplex polymerase chain reaction (PCR) and capillary gel electrophoresis for microsatellite sites. PCR-based MSI testing is the golden standard method for detecting MSI status, which is determined by visual assessment of allele size changes. The National Cancer Institute recommended to use Bethesda panel with five markers, which includes two mononucleotide repeats of BAT-25 and three dinucleotide repeats of D5S346, D2S123, and D17S250 (8). To compare the length of microsatellite markers between tumor and matched normal sample, we measure the length of those markers to determine the MSI status of a tumor sample. Tumors with altered lengths of two or more markers, one marker, and zero markers are classified as high MSI (MSI-H), low MSO (MSI-L), and microsatellite stable (MSS), respectively. An alternative PCR-based MSI testing panel relies on five poly-A mononucleotide repeats (BAT-25, BAT-26, NR-21, NR-24, and NR-27). It is considered more standard because of its high specificity and sensitivity (9). Another panel containing six mononucleotide repeats (NR-27, NR-28, Bat-25, Bat-26, NR-24, and Mono-27) and three pentanucleotide markers (Penta C, Penta D, and Amel) is also used to detect MSI status (10).

IHC-based MMR detection method is practicable and cost-effective. The loss of IHC expression of an MMR protein reveals the status of a specific target gene in the confirmatory testing. However, accurate interpretation of staining results requires well-trained pathologists. Furthermore, some MMR gene mutations may produce dysfunctional proteins and IHC stain expression, which leads to a false positive result (11, 12).

Because tissue samples from patients are limited, it is important to improve the efficiency of the testing. Targeted next-generation sequencing (NGS) has brought an unprecedented scale of genomic data, allowing dozens to hundreds of genes to be sequenced simultaneously and with higher sensitivity for low-prevalence mutations (13). The main advantage of using NGS to test MSI status is the ability to determine tumor mutation burden (TMB), along with other potential targetable alterations simultaneously. Therefore, NGS-based algorithms have emerged as a new way to detect MSI status. Several NGS-based MSI detection methods have been proposed in recent years (13–15). NCCN recommends to detect MSI and TMB and genes associated with targeted therapy at the same time. NGS-based methods can accomplish these tasks in one single run, but PCR-based method cannot.

Combining NGS with analysis tools, such as MSIsensor, one can reliably infer MSI status from large-panel targeted NGS data (14). Here, we developed a new algorithm to detect MSI status based on NGS data. Moreover, our algorithm supplied the unstable markers in an explainable way, which might bring new insights into the therapies for cancer patients. Since currently, clinical research tends to combine MSS-L and MSS as one single status (16), we classify our samples into only MSI-H and MSI-L samples.

## Results

### Sample Collection

A total of 178 samples were collected from Chifeng Municipal Hospital. The matched clinical data are summarized as **Table 1** and detailed information could be found in **Supplementary Table 2**. Both tumor and matched blood sample were sequenced for all patients. This dataset was split by chronological order into a training set (nine samples), a validation set (101 samples), and a test set (68 samples). During the hyperparameter tuning phase, only the training set could be accessed by the algorithm. The validation set was used to estimate the algorithm’s accuracy. Once the hyperparameters were optimized, the test set was used to evaluate the accuracy and generalization ability of the proposed algorithm.

**Table 1:** Summary for samples.

Number of Samples	MSI-L	MSI-H	Total
Training set	9	0	9
Validation set	92	9	101
Test set	65	3	68
Total	166	12	178

**Figure 1:**
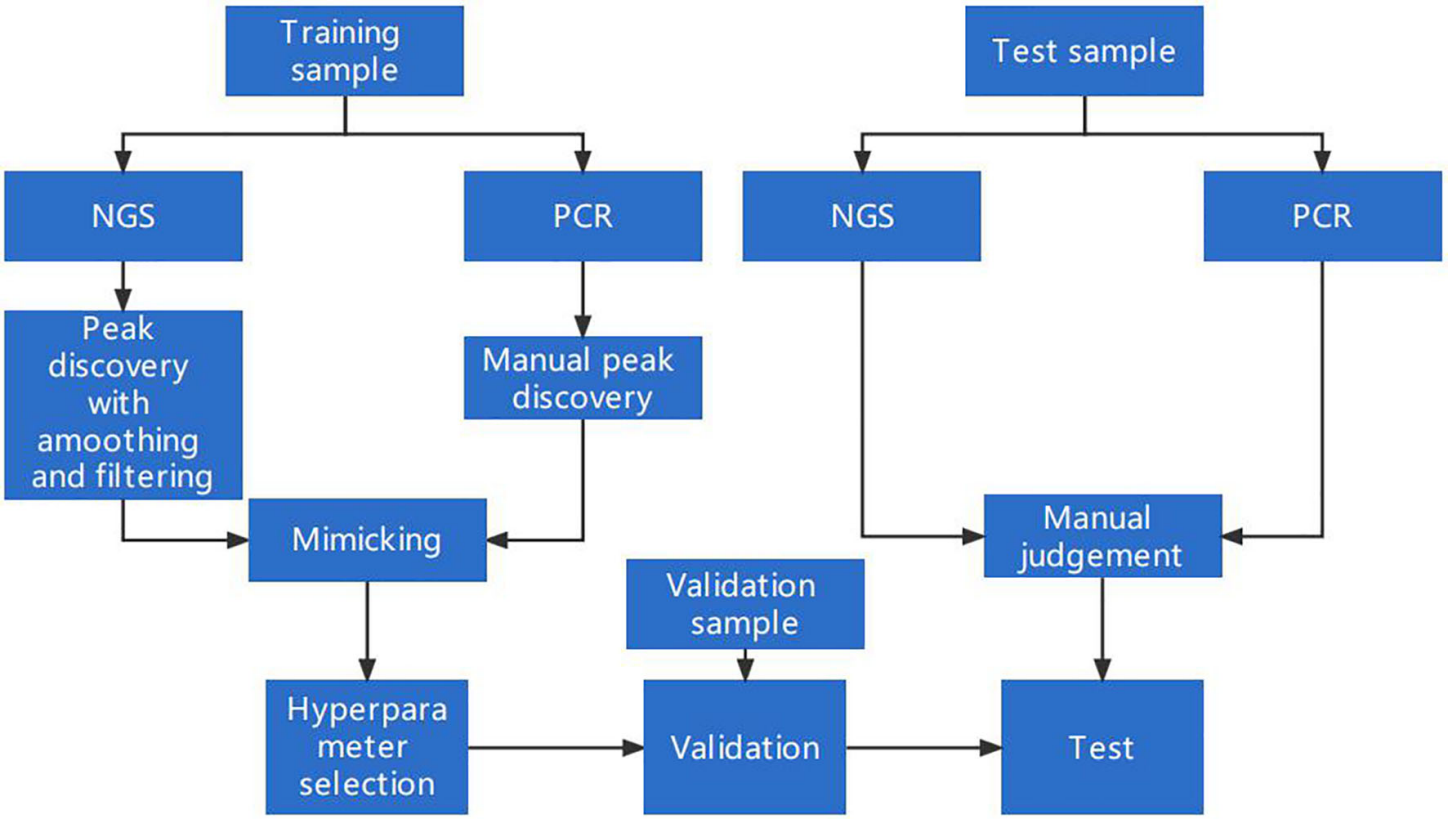
A schematic for MSI detection and algorithm optimization. The dataset was split into three parts: a training set, a validation set, and a test set. The training set was used to design and optimize the peak-finding algorithm in the NGS data to mimic the PCR result. The validation set was used for determining whether the algorithm could be generalized to samples beyond the training set. The test set was collected independently to further evaluate the algorithm’s performance.

A schematic of our algorithm is shown in **Figure 1**.

### Peaks From NGS Data Were Similar to PCR Results

According to the PCR-based method, the numbers of peaks could be inferred to evaluate the MSI status. Thus, we designed an algorithm to locate the peaks in the genome to make the peak numbers inferred by the NGS-based method are close to that number generated by the PCR-based method.

**Figure 2:**
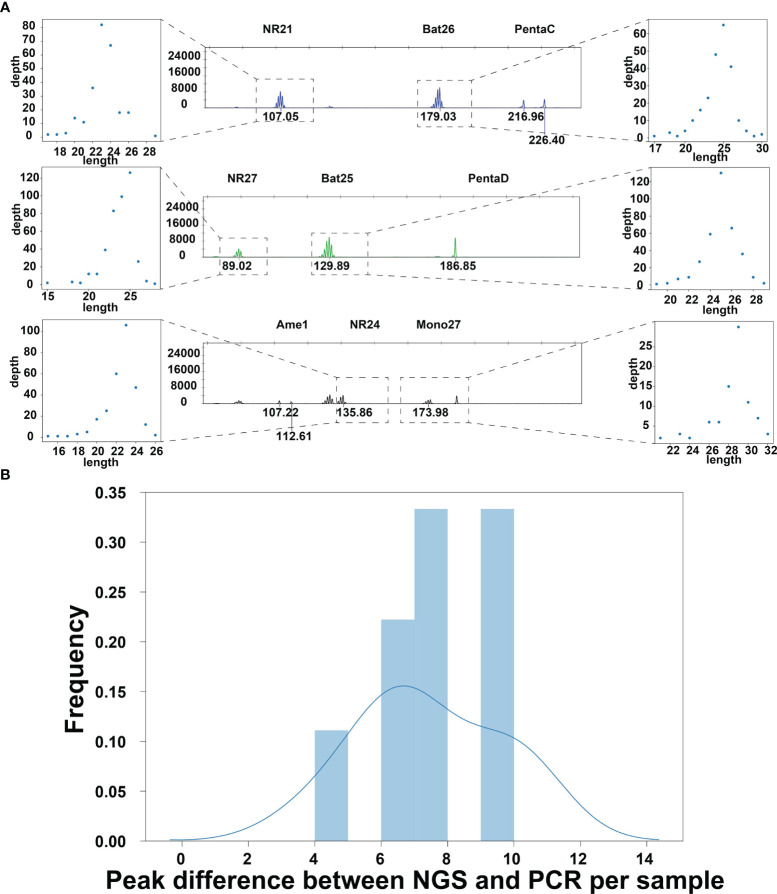
**(A)** The peaks detected by the PCR method and the NGS method in the tumor sample of 21CF30228. The peaks detected in the PCR data were shown in the middle [the x-axis is the PCR product length, and the y-axis is the corresponding Relative Fluorescence Units (RFU)], and the peaks detected in the NGS data were plotted accordingly on the left and right. As shown in the figure, the peaks were similar between the NGS data and the PCR data. **(B)** The peak discovered from the raw depth plot has a huge difference between NGS data and PCR data. The peaks’ lengths detected by the PCR are larger than that detected by NGS. This is due to the shift caused by PCR primer not being adjacent to the repeat sequence of the markers.

Our method first extracts read depth information from the input BAM file, such depth information is visualized as **Figure 2A**. We then calculated the peak numbers for NGS data and compared them with matched PCR results. The results show that, without any additional manipulations, the peaks discovered in NGS data are more than those identified in the PCR result (from 4 to 10 for all samples in the training set, **Figure 2B**). Peaks that were only detected in the NGS data are potential technical noise in the sequencing data caused by diminishing of light strength linearity (17). Thus, noise reduction is needed for an accurate peak detection method developed for the NGS data.

### Restricting peak height and smoothing is helpful for noise removal in NGS data.

**Figure 3:**
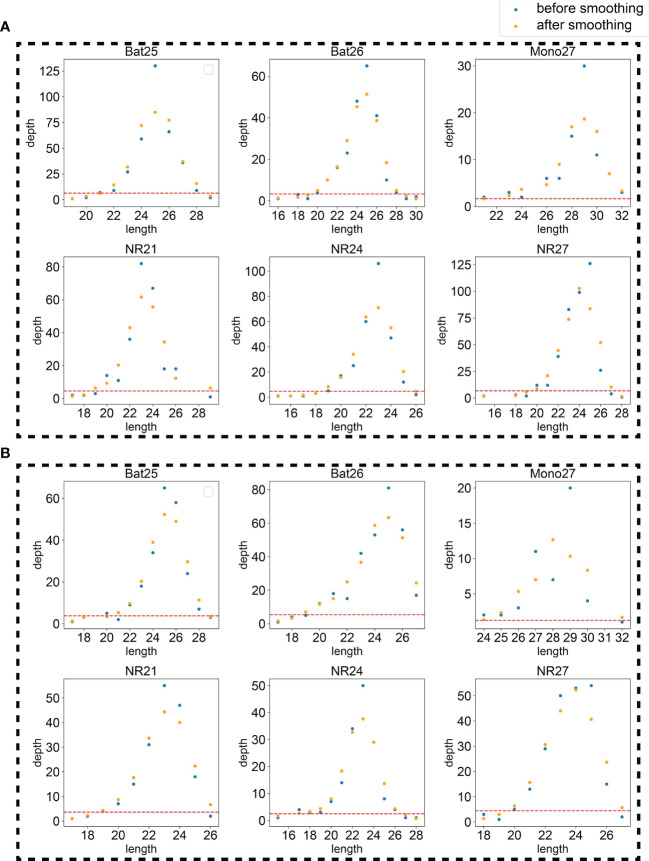
An example using the tumor sample **(A)** and normal sample **(B)** of 21CF30228 to display the effects of the two hyperparameters, smoothing and peak threshold restricting. The red line on the bottom of each figure is the threshold for peak filtering. Peaks detected under the threshold will be ignored.

Because the NGS data might be noisy, two strategies, restricting peak height and smoothing, were used to eliminate false peaks. Restricting peak height is to remove peaks with too low heights (see Materials and methods for details). The height threshold was set as a hyperparameter for further tuning. An example applying the hyperparameters was plotted in **Figure 3** to intuitively display the effects of the two hyperparameters.

The goal of smoothing is to eliminate the noise caused by sequencing errors and to capture true signals. Smoothing or not was set as candidate procedures for further hyperparameter determination.

### Hyperparameter Determination

After creating a set of parameters, we defined a loss function *diff* (see Loss function to minimize section in Materials and methods) to minimize the difference between the actual peak numbers that detected by PCR method and our algorithm.

**Figure 4:**
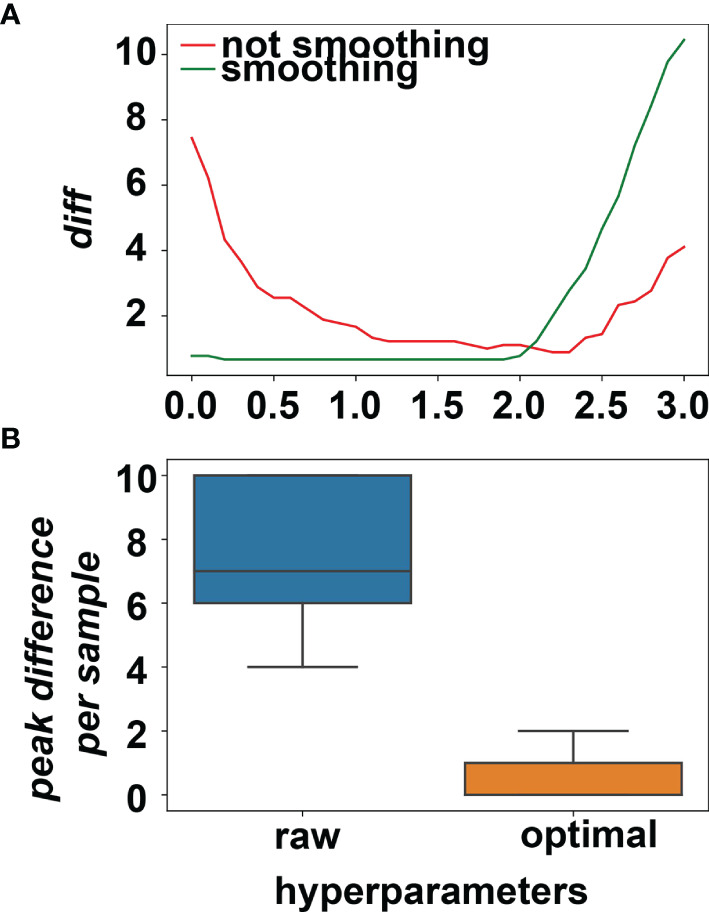
**(A)** The average difference between NGS data and PCR data changes for different hyperparameters. The minimum of average difference was obtained when smoothing was applied and the peak threshold was set to (20% × average depth). **(B)** The peak differences between NGS and PCR for all the training samples before and after picking the optimal hyperparameters.

We performed grid search for the optimal hyperparameters: whether smoothing or not combining with the peak height threshold among 0.1, 0.2, 0.3, …, 3.0. *diff* values were plotted against the peak height threshold and smoothing or not in **Figure 4A**. The minimum *diff* value of the smoothing method is 0.66, whereas the minimum *diff* of the non-smoothing method is 0.88. Because a lower threshold guarantees more sensitivity, the hyperparameters were set as smoothing and 0.2 for peak height threshold. The differences of the peaks detected in the NGS and PCR for per sample were plotted in **Figure 4B**, showing the algorithm under the optimal hyperparameters could sufficiently mimic the PCR result.

### The Algorithm Performed Well on the Independent Dataset

The optimized hyperparameters were applied to our algorithm to find peaks in the NGS data. After peak discovery, the six markers for each patient were determined as stable or unstable using the same standards of the PCR-based method, which would finally result in the classification of MSI status for the patients (see Patient MSI status determination section of Materials and methods for details).

Finally, we achieved a 100% accuracy for the validation dataset and 98.53% accuracy for the test dataset, in which only one false positive result was reported. This indicates that our algorithm is applicable for clinical diagnosis. We also calculated the recall and specificity for all cancers for both validation set and test set. In most cancers, 100% accuracy was achieved. All metrics were summarized in **Supplementary Table 3**.

### Comparisons to MSIsensor

**Figure 5:**
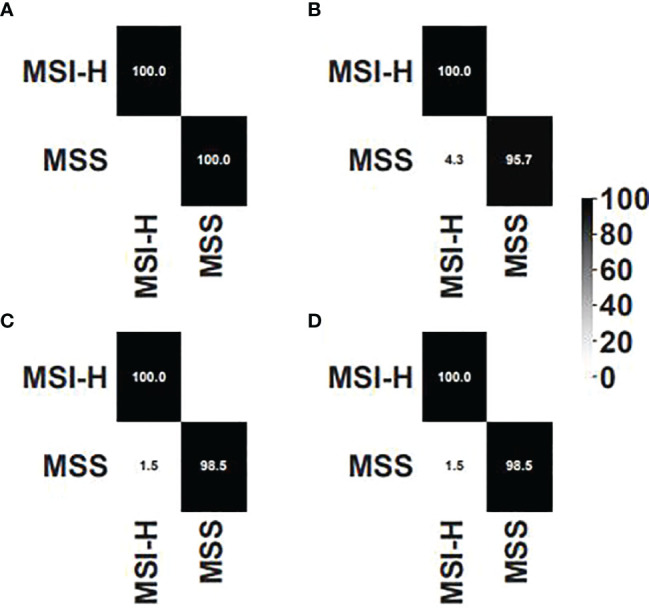
Confusion matrix for **(A)** our algorithm on the validation set, **(B)** MSIsensor on the validation set, **(C)** our algorithm on the test set, and **(D)** MSIsensor on the test set.

We compared our algorithm to another MSI detection algorithm, MSIsensor, which is currently one of the best algorithms for MSI detection. The same training set, validation set, and test set were used to choose the threshold for MSIsensor. Three thresholds, i.e., 3.5%, 20%, and 40%, were chosen as the candidate. As for the threshold of 3.5%, there was one false positive in the training dataset. As for the threshold of 20%, there were 7 false positives, whereas only four false positives were detected for the threshold of 40%. Thus, 40% was set to the threshold for MSIsensor. The performances of our method and MSIsensor were plotted in confusion matrix in **Figure 5**. As shown in the validation set, our algorithm outperformed MSIsensor. However, they were equally accurate in the test set. One false positive was detected by our algorithm and MSIsensor separately. However, our algorithm detected 21CF30073 as a false positive and MSIsensor detected 20CF30697 as a false positive. Furthermore, as shown in **Supplementary Table 2**, if we combine the two methods and determine MSI-H only if the two methods both determined MSI-H for one sample, then 100% accuracy would be achieved.

## Materials and Methods

### PCR Method to Determine MSI Status

The Microread MSI Kit was used to determine the MSI status. First the six pairs of primers were used to amplify both tissues from tumor and matched blood as normal. Capillary electrophoresis was then used to genotype the lengths of the products amplified by the primers. GeneMapper was finally used to check the peaks manually to assess whether the tumor tissue consists of unstable markers compared to the matched normal tissue.

### Next-Generation Sequencing

We chose six markers covered by Microread MSI Kit for further research. The corresponding probes for each marker were listed in **Supplementary Table 1**.

The capture probes were designed and produced by BOKE Co., Ltd. The samples were prepared for targeted NGS using BGI sequencer MGISEQ-2000.

### Pipeline for Depth Extraction

To simulate the results from PCR-based method, a histogram of count for each length is needed. A regular bioinformatics pipeline was first used for BAM file generation. First, trimmomatic v0.38 was used to trim adapters. Second, bwa v0.7.12 aln was used for alignment using hg19 as the reference genome. SAMtools v0.1.19 sort and rmdup were applied to the BAM file. For each marker, the surrounding reads were extracted from bam file using SAMtools v0.1.19. Afterward, using the anchor sequence, we calculated the read number for all markers. The position and the anchor sequence for all markers are listed in **Table 2**. For each marker, the reads were extracted from BAM file using SAMtools v0.1.19 and then matched with the anchor regex or complementary reverse anchor regex to count for the marker sequences. All anchor sequences were confirmed to be uniquely mapped to the sequencing within the scope of that marker’s position. The script CollectDepthInfoForMSI.sh is for collecting the depths for each length from the BAM file.

**Table 2:** Anchor sequences for each marker for obtaining peak. CR, complementary reverse.

Marker	Position	Anchor	Anchor (CR)	Repeat Length
Bat25	chr4:55598012-55598436	TTTGA(T+)GAGAA	TTCTC(A+)TCAAA	25
Bat26	chr2:47641360-47641786	CAGGT(A+)GGGTT	AACCC(T+)ACCTG	27
Mono27	chr2:39536490-39536916	CAGGA(T+)GAGGC	GCCTC(A+)TCCTG	27
NR21	chr14:23652147-23652567	TTGCT(A+)GGCCA	TGGCC(T+)AGCAA	21
NR24	chr2:95849162-95849585	TCCTA(T+)GTGAG	CTCAC(A+)TAGGA	23
NR27	chr11:102193309-102193734	CTGGT(A+)GCCAC	GTGGC(T+)ACCAG	26

### Peak Discovery

We define eminent number in the middle of three numbers as a peak. For recalling the peaks at the end of a series of numbers, we added zeros at the start and the end of the series of peaks to make sure that the first and the last number could also be detected as a peak.

### Smoothing and De-Duplication

Our algorithm is a peak finding algorithm in the noised NGS data. Thus, we implemented the robust peak discovery algorithm, attempting to avoid discovering false peaks. The main strategy for noise reduction is smoothing procedure, which involves calculating the average height among flanks.

To remove the noise that might be caused by experimental artifacts, the following procedures were applied.

First, the relative height was calculated to standardize the heights and the relatively low peaks were recognized as false peaks. The relative height of the *n*th position is defined as


$$rh_{n}=\frac{h_{n}}{\sum_{i=1}^{N}h_{i}}$$

where *N* is the total number of detected peaks and *h* is the absolute height of each peak. A hyperparameter, *R_h_*, is set as the threshold for peaks. If a peak is discovered and its *rh*_*n*_≥*R*_*h*_, then the peak is reckoned as a real peak; otherwise, the peak is considered as a false peak caused by technical noise. This step will help eliminate some false peaks.

Second, the smoothing step was applied. Smoothing step is a procedure to calculate the average height among its neighborhood:


$$h_{n}=\frac{h_{n-1}+h_{n}+h_{n+1}}{3}$$

### Unstable Marker Discovery

After peak discovery, the markers could be determined as stable or unstable according to the comparison between tumor and normal samples. If a marker has different peak numbers in tumor and normal tissue, then the marker would be classified as an unstable marker. If the peak numbers are the same for both tissues, then a gap ≥2 for tumor/normal tissue peak position would lead to an unstable marker judgment.

### Loss Function to Minimize

To minimize the gap between NGS data and PCR data, a loss function is defined as


$$diff=\;\frac{\sum_{i}^{S}\sum_{t}^{T}abs\left(P_{NGS}^{i,t}-P_{PCR}^{i,t}\right)}{S}$$

where *S* is the total number samples, *T* is the total number of markers, $P_{NGS}^{i,t}$ is the number of peaks discovered in the *i*th sample in the *t*th marker in NGS data by the algorithm, and $P_{PCR}^{i,t}$ is the peak number discovered in the *i*th sample in the *t*th marker in the PCR data. This loss function could evaluate the average difference in peaks between NGS data and PCR data for each sample.

### Patient MSI Status Determination

After determination of all six makers for a patient, the MSI status for a patient could be determined. For a patient with two or more unstable markers, we would regard the patient as a patient with MSI-H. This is consistent with the determination standard of PCR-based method. The script JudgeMSI.py is for determining the MSI status according to the depth files from tumor and normal samples.

### Detection by MSIsensor

The BAM file was prepared as mentioned above, and the bed for the six markers were used. The other parameters were set to default.

## Discussion

In this work, we implemented a novel algorithm to determine MSI status based on NGS data. Our algorithm could simulate the distribution of peaks. In the independent 68-sample dataset, we achieved an accuracy of 98.53% and a sensitivity of 100%, showing promise in clinical practice. Furthermore, very small number of samples is sufficient to train an accurate model. As shown in our work, we used only nine samples as the training set. In fact, there are 108 markers for our hyperparameter tuning. According to the accuracy in the validation set and the test set, the small amount of training set was enough for an accurate mimicking from NGS to the PCR result. Furthermore, because the MSI status is highly imbalanced with few MSI-H samples, our method requiring no MSI-H samples is an advantage. Our method also outperformed MSIsensor on our dataset, and we also found that combining the two methods might make the result more accurate. Because the false negative for MSI detection is severer, this finding should be verified by more data.

Further efforts could be made for the development of the algorithm. First of all, more data are needed to generalize the hyperparameters for more real-world data and data from other platforms such as Illumina. As a pan-cancer dataset, our work also needs more data for validation, because many cancers were underrepresented in our dataset. More peak discovery hyperparameters could be involved such as distance and prominence (18). Recently, machine learning is emerging as a handy tool for bioinformatics in both biological applications (19–24) and theory discovery (25–28). Because this work is data-driven, machine learning could be also used to address it. However, most machine learning algorithms require input of uniform length, which is not satisfied by NGS data. Thus, we need to design methods to generate uniform features from diverse NGS data. On the other hand, recurrent neural network (RNN), which has been vastly used for natural language processing (29), could solve the problem with inconsistent features and has been applied to biology years ago (30). The RNN has advantages over handcrafted features and might be applicable to this problem.

## Conclusion

Here, we developed an algorithm to determine MSI status for patients with cancer using the NGS data, and the accuracy is comparable to the gold standard, PCR-based method. The accuracy and the sensitivity are both acceptable for clinical use.

## Data Availability Statement

The data presented in the study are deposited in the NCBI BioProject repository, accession number PRJNA810563.

## Ethics Statement

The studies involving human participants were reviewed and approved by Chifeng Municipal Hospital, Inner Mongolia, China. The patients/participants provided their written informed consent to participate in this study.

## Author Contributions

BW contributed to the conception and design of the study. SL, LT, and MC organized the database. BW and RH performed the statistical analysis. SL and LT wrote the first draft of the manuscript. RH and GT revised the manuscript. All authors contributed to the article and approved the submitted version.

## Funding

This study was partially supported by the Natural Science Foundation of Inner Mongolia Autonomous Region of China (Grant No. 2019MS08187).

## Conflict of Interest

The authors RH, GT, and BW are employed by Genesis (Beijing) Co., Ltd.

The remaining authors declare that the research was conducted in the absence of any commercial or financial relationships that could be construed as a potential conflict of interest.

## Publisher’s Note

All claims expressed in this article are solely those of the authors and do not necessarily represent those of their affiliated organizations, or those of the publisher, the editors and the reviewers. Any product that may be evaluated in this article, or claim that may be made by its manufacturer, is not guaranteed or endorsed by the publisher.

## Supplementary Material

Supplementary Table 1 Marker information.

Supplementary Table 2 Clinical diagnosis of patients and the results of our algorithm and MSIsensor.

Supplementary Table 3 Metrics for different datasets and different cancers.

## References

[1] Goel A Boland CR. Epigenetics of Colorectal Cancer. Gastroenterology (2012) 143(6):1442–60.e1441. doi: 10.1053/j.gastro.2012.09.032 23000599 PMC3611241

[2] Latham A Srinivasan P Kemel Y Shia J Bandlamudi C Mandelker D. Microsatellite Instability Is Associated With the Presence of Lynch Syndrome Pan-Cancer. J Clin Oncol (2019) 37(4):286–95. doi: 10.1200/jco.18.00283 PMC6553803 30376427

[3] Carethers JM Smith EJ Behling CA Nguyen L Tajima A Doctolero RT. Use of 5-Fluorouracil and Survival in Patients With Microsatellite-Unstable Colorectal Cancer. Gastroenterology (2004) 126(2):394–401. doi: 10.1053/j.gastro.2003.12.023 14762775

[4] Fuchs CS Doi T Jang RW Muro K Satoh T Machado M. Safety and Efficacy of Pembrolizumab Monotherapy in Patients With Previously Treated Advanced Gastric and Gastroesophageal Junction Cancer: Phase 2 Clinical KEYNOTE-059 Trial. JAMA Oncol (2018) 4(5):e180013. doi: 10.1001/jamaoncol.2018.0013 29543932 PMC5885175

[5] Liu H Qiu C Wang B Bing P Tian G Zhang X. Evaluating DNA Methylation, Gene Expression, Somatic Mutation, and Their Combinations in Inferring Tumor Tissue-Of-Origin. Front Cell Dev Biol (2021) 9:619330. doi: 10.3389/fcell.2021.619330 34012960 PMC8126648

[6] Overman MJ Lonardi S Wong KYM Lenz HJ Gelsomino F Aglietta M. Durable Clinical Benefit With Nivolumab Plus Ipilimumab in DNA Mismatch Repair-Deficient/Microsatellite Instability-High Metastatic Colorectal Cancer. J Clin Oncol (2018) 36(8):773–9. doi: 10.1200/jco.2017.76.9901 29355075

[7] Overman MJ McDermott R Leach JL Lonardi S Lenz HJ Morse MA. Nivolumab in Patients With Metastatic DNA Mismatch Repair-Deficient or Microsatellite Instability-High Colorectal Cancer (CheckMate 142): An Open-Label, Multicentre, Phase 2 Study. Lancet Oncol (2017) 18(9):1182–91. doi: 10.1016/s1470-2045(17)30422-9 PMC6207072 28734759

[8] Umar A Boland CR Terdiman JP Syngal S de la Chapelle A Rüschoff J. Revised Bethesda Guidelines for Hereditary Nonpolyposis Colorectal Cancer (Lynch Syndrome) and Microsatellite Instability. J Natl Cancer Inst (2004) 96(4):261–8. doi: 10.1093/jnci/djh034 PMC2933058 14970275

[9] Goel A Nagasaka T Hamelin R Boland CR. An Optimized Pentaplex PCR for Detecting DNA Mismatch Repair-Deficient Colorectal Cancers. PLoS One (2010) 5(2):e9393. doi: 10.1371/journal.pone.0009393 20195377 PMC2827558

[10] Zhang W Yin H Huang Z Zhao J Zheng H He D. Development and Validation of MRI-Based Deep Learning Models for Prediction of Microsatellite Instability in Rectal Cancer. Cancer Med (2021) 10(12):4164–73. doi: 10.1002/cam4.3957 PMC8209621 33963688

[11] Hechtman JF Rana S Middha S Stadler ZK Latham A Benayed R. Retained Mismatch Repair Protein Expression Occurs in Approximately 6% of Microsatellite Instability-High Cancers and is Associated With Missense Mutations in Mismatch Repair Genes. Mod Pathol (2020) 33(5):871–9. doi: 10.1038/s41379-019-0414-6 PMC7195218 31857677

[12] McCarthy AJ Capo-Chichi JM Spence T Grenier S Stockley T Kamel-Reid S. Heterogenous Loss of Mismatch Repair (MMR) Protein Expression: A Challenge for Immunohistochemical Interpretation and Microsatellite Instability (MSI) Evaluation. J Pathol Clin Res (2019) 5(2):115–29. doi: 10.1002/cjp2.120 PMC6463865 30387329

[13] Salipante SJ Scroggins SM Hampel HL Turner EH Pritchard CC. Microsatellite Instability Detection by Next Generation Sequencing. Clin Chem (2014) 60(9):1192–9. doi: 10.1373/clinchem.2014.223677 24987110

[14] Middha S Zhang L Nafa K Jayakumaran G Wong D Kim HR. Reliable Pan-Cancer Microsatellite Instability Assessment by Using Targeted Next-Generation Sequencing Data. JCO Precis Oncol (2017)2017 1–17. doi: 10.1200/po.17.00084 PMC6130812 30211344

[15] Niu B Ye K Zhang Q Lu C Xie M McLellan MD. MSIsensor: Microsatellite Instability Detection Using Paired Tumor-Normal Sequence Data. Bioinformatics (2014) 30(7):1015–6. doi: 10.1093/bioinformatics/btt755 PMC3967115 24371154

[16] Li K Luo H Huang L Luo H Zhu X. Microsatellite Instability: A Review of What the Oncologist Should Know. Cancer Cell Int (2020) 20:16. doi: 10.1186/s12935-019-1091-8 31956294 PMC6958913

[17] Ivady G Madar L Dzsudzsak E Koczok K Kappelmayer J Krulisova V. Analytical Parameters and Validation of Homopolymer Detection in a Pyrosequencing-Based Next Generation Sequencing System. BMC Genomics (2018) 19(1):158. doi: 10.1186/s12864-018-4544-x 29466940 PMC5822529

[18] Virtanen P Gommers R Oliphant TE Haberland M Reddy T Cournapeau D. SciPy 1.0: Fundamental Algorithms for Scientific Computing in Python. Nat Methods (2020) 17(3):261–72. doi: 10.1038/s41592-019-0686-2 PMC7056644 32015543

[19] He B Dai C Lang J Bing P Tian G Wang B. A Machine Learning Framework to Trace Tumor Tissue-of-Origin of 13 Types of Cancer Based on DNA Somatic Mutation. Biochim Biophys Acta Mol Basis Dis (2020) 1866(11):165916. doi: 10.1016/j.bbadis.2020.165916 32771416

[20] Liang X Zhu W Liao B Wang B Yang J Mo X. A Machine Learning Approach for Tracing Tumor Original Sites With Gene Expression Profiles. Front Bioeng Biotechnol (2020) 8:607126. doi: 10.3389/fbioe.2020.607126 33330438 PMC7732438

[21] Meng Y Lu C Jin M Xu J Zeng X Yang J. A Weighted Bilinear Neural Collaborative Filtering Approach for Drug Repositioning. Brief Bioinform (2022) 23(2):bbab581. doi: 10.1093/bib/bbab581 35039838

[22] Peng L Peng M Liao B Huang G Dingfeng X. The Advances and Challenges of Deep Learning Application in Biological Big Data Processing. Curr Bioinf (2018) (8):352–9. doi: 10.2174/1574893612666170707095707

[23] Yang J Ju J Guo L Ji B Shi S Yang Z. Prediction of HER2-Positive Breast Cancer Recurrence and Metastasis Risk From Histopathological Images and Clinical Information via Multimodal Deep Learning. Comput Struct Biotechnol J (2022) 20:333–42. doi: 10.1016/j.csbj.2021.12.028 PMC8733169 35035786

[24] Liang Y Wang H Yang J Li X Dai C Shao P. A Deep Learning Framework to Predict Tumor Tissue-Of-Origin Based on Copy Number Alteration. Front Bioeng Biotechnol (2020) 8:701. doi: 10.3389/fbioe.2020.00701 32850687 PMC7419421

[25] Peng L Liu F Yang J Liu X Meng Y Deng X. Probing lncRNA-Protein Interactions: Data Repositories, Models, and Algorithms. Front Genet (2019) 10:1346. doi: 10.3389/fgene.2019.01346 32082358 PMC7005249

[26] Peng L Peng M Liao B Xiao Q Liu W Huang G. A Novel Information Fusion Strategy Based on a Regularized Framework for Identifying Disease-Related microRNAs. RSC Adv (2017) 7(70):44447–55. doi: 10.1039/C7RA08894A

[27] Xu J Cai L Liao B Zhu W Yang J. CMF-Impute: An Accurate Imputation Tool for Single-Cell RNA-Seq Data. Bioinformatics (2020) 36(10):3139–47. doi: 10.1093/bioinformatics/btaa109 32073612

[28] Zhou L Li Z Yang JA-O Tian G Liu F Wen H. Revealing Drug-Target Interactions With Computational Models and Algorithms. Molecules (2019) 27(9):1–36. doi: 10.3390/molecules24091714 PMC6540161 31052598

[29] Lerner I Paris N Tannier X. Terminologies Augmented Recurrent Neural Network Model for Clinical Named Entity Recognition. J BioMed Inform (2020) 102:103356. doi: 10.1016/j.jbi.2019.103356 31837473

[30] Min S Lee B Yoon S. Deep Learning in Bioinformatics. Brief Bioinform (2016) 18(5):851–69. doi: 10.1093/bib/bbw068 27473064

